# Fluorescent labeling of dendritic spines in cell cultures with the carbocyanine dye “DiI”

**DOI:** 10.3389/fnana.2014.00030

**Published:** 2014-05-09

**Authors:** Connie Cheng, Olivia Trzcinski, Laurie C. Doering

**Affiliations:** Department of Pathology and Molecular Medicine, McMaster UniversityHamilton, ON, Canada

**Keywords:** DiI, carbocyanine dye, dendritic spine, morphology, confocal microscopy, paraformaldehyde, neuronal function

## Abstract

Analyzing cell morphology is a key component to understand neuronal function. Several staining techniques have been developed to facilitate the morphological analysis of neurons, including the use of fluorescent markers, such as DiI (1,1′-dioctadecyl-3,3,3′,3′-tetramethylindocarbocyanine perchlorate). DiI is a carbocyanine membrane dye that exhibits enhanced fluorescence upon insertion of its lipophilic hydrocarbon chains into the lipid membrane of cells. The high photostability and prominent fluorescence of the dye serves as an effective means of illuminating cellular architecture in individual neurons, including detailed dendritic arborizations and spines in cell culture and tissue sections. Here, we specifically optimized a simple and reliable method to fluorescently label and visualize dissociated hippocampal neurons using DiI and high-resolution confocal microscopic imaging. With high efficacy, this method accurately labels neuronal and synaptic morphology to permit quantitative analysis of dendritic spines. Accurate imaging techniques of these fine neuronal specializations are vital to the study of their morphology and can help delineate structure-function relationships in the central nervous system.

## INTRODUCTION

**FIGURE 1:**
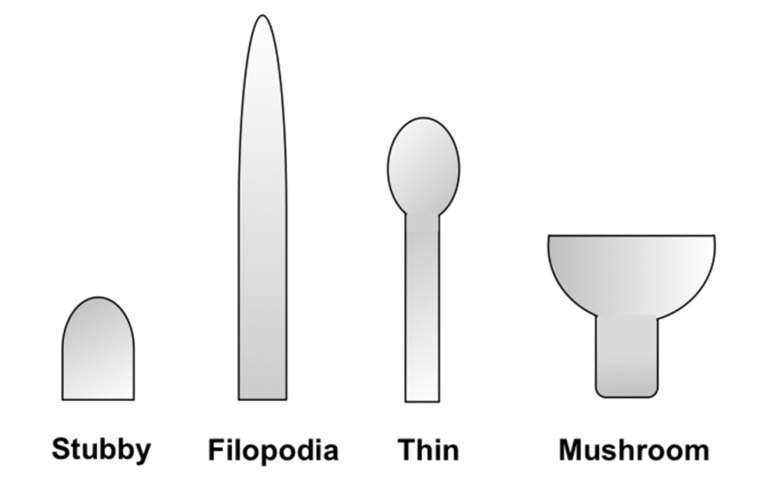
**Schematic representation of spine morphologies.** Spines display a wide diversity of morphologies. They are commonly classified into four different categories (as illustrated from left to right): stubby, filopodia, thin, and mushroom-shaped. Stubby spines are devoid of a neck and are particularly prominent during postnatal development. Thin spines are most common and have a thin, long neck and a small bulbous head, whereas mushroom spines are those with a large head. Lastly, dendritic filopodia are typically longer, normally have no clear head, and often represent immature spines.

Dendritic spines are small protrusions from the dendritic shaft of various types of neurons that act as the postsynaptic compartments of most excitatory synapses in the central nervous system (CNS). They are known to play a significant role in neuronal plasticity and synaptic integration through their ability to undergo structural rearrangements during development (Rochefort and Konnerth, 2012). Morphological features of spines, such as size, shape, and density, have been shown to reflect important synaptic functional attributes and the potential for plasticity. Spine morphology is highly variable and has been classified into several different types based on their structure: filopodia, long-thin, stubby, and mushroom-shaped (Yuste, 2011; **Figure 1**). On the same dendrite, a continuum of shapes can be observed and the morphology can change rapidly through activity-dependent and -independent mechanisms (Penzes and Rafalovich, 2012). The density of spines can be understood in terms of the levels of connectivity within the neuronal network, as well as the integrative capabilities of the neuron. As such, abnormalities in the shape and density of spines can often signify an aspect of disease (Fiala et al., 2002). Therefore, structural classifications of spines with accurate labeling and imaging techniques can spawn vital information on neuronal function, and in turn offer insight into the etiology of neurological diseases (Lin and Koleske, 2010; Penzes et al., 2011).

For over a century, the Golgi staining technique has been the classical method for neuronal labeling and dendritic spine analysis (Neely et al., 2009). Although Golgi staining has played a crucial role in the advancement of anatomical neurobiology, a major drawback of this technique is that the tissue fixation used for Golgi is often incompatible with other methods to study morphology, such as immunocytochemistry. Furthermore, the Golgi method provides inconsistent, low frequency staining, which results in the insecurity of a selection bias (Staffend and Meisel, 2011b). In addition, long periods of time (often weeks) are required to reach the final product. Due to the lack of specificity and reproducibility, researchers have decreasingly relied on this technique (Ranjan and Mallick, 2010). A variety of methods have been developed to circumvent some of the limitations of the Golgi staining, most notably through the use of fluorescent markers. In fact, labeling cells and tissues with fluorescent markers is one of the most widely used methods of cellular examination employed to date (Colello et al., 2012). Fluorescence immunolabeling is a highly specific method that is commonly used to visualize cell structure, facilitate protein localization, and study cell interactions at the light microscopic level. Some of these other methods used to evaluate cellular morphology include various commercially available dyes, fluorochrome labeled antibodies, and genetically encoded fluorescent proteins, such as green fluorescent protein (GFP; Staffend and Meisel, 2011a). Specifically with GFP, transgenic animals and cultured cells can be designed to drive fluorescent expression under specific promoters (Malinow et al., 2010). However, although GFP-labeling provides great specificity of fluorescent expression, a similar end result can be accomplished in a much shorter time frame and with far fewer supplies/materials by employing lipophilic DiI labeling.

**FIGURE 2:**
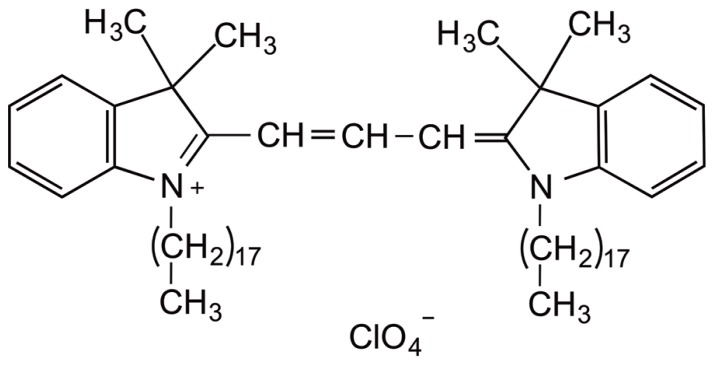
**Chemical structure of carbocyanine dye DiI.** The fluorescent lipophilic dye “DiI” [1,1′-dioctadecyl-3,3,3′,3′ tetramethylindocarbocyanine perchlorate; DiIC18(3)] is commonly used as an anterograde and retrograde neuronal tracer. DiI labels cell membranes by inserting its two long (C_18_ carbon) hydrocarbon chains into the lipid bilayers.

The fluorescent lipophilic dye dialkylcarbocyanine, also called “DiI” [1,1′-dioctadecyl-3,3,3′,3′-tetramethylindocarbocyanine perchlorate; DiIC_18_(3)], has traditionally been used for anterograde and retrograde neuronal tracing (Honig and Hume, 1989). Structurally, the molecule consists of a hydrophilic head that lies above the plasma cell membrane and two lipophilic hydrocarbon side chains that insert into the hydrophobic plasma membrane (Bruce et al., 1997; **Figure 2**). The orange-red fluorescent dye is weakly fluorescent until it is incorporated into the membrane. DiI partitions and diffuses through the cell membrane to sufficiently highlight dendrites and their spinous protrusions, providing a well-defined outline of neuronal processes (Sherazee and Alvarez, 2013). The fluorescence provided by the carbocyanine dye is very strong and robust and withstands illumination, e.g., in a confocal laser scanning microscope (Gan et al., 1999; Lanciego and Wouterlood, 2011). Typical applications of this technique include the study of neuronal morphology during development and altered development in neurological disorders (Bruce et al., 1997; Braun and Segal, 2000; Smith et al., 2009; Li et al., 2010). This dye can be applied to a variety of cell types, live or fixed tissue (Terasaki et al., 1994), as well as diverse species such as rodents, primates, and zebrafish (Gan et al., 2000; O’Brien and Lummis, 2006; Seabold et al., 2010; Arsenault and O’Brien, 2013). In slice preparations, DiI labeling is commonly known as “DiOlistic labeling,” in which beads coated with the lipophilic dye are “ballistically” ejected with a gene gun on to brain tissue (Lo et al., 1994). This technique has been developed as a useful and simple means to label neurons and glia in their entirety, unveiling even the most detailed structures, such as dendritic spines (Haber et al., 2006). To date, very few procedures are available that allow the direct application of DiI to cultured cells. Our protocol described here results in high quality staining and imaging of dissociated cell cultures with lipophilic DiI labeling and confocal microscopy. This visualization approach enables a detailed analysis of dendritic spine morphology, density, topographical distribution, and connectivity.

## MATERIALS AND METHODS

### ANIMALS

An in-house mouse breeding colony was used to generate primary cell cultures of hippocampal neurons for DiI labeling. The mice were housed and bred at the McMaster University Central Animal Facility. All experiments complied with the guidelines set out by the Canadian Council on Animal Care and were approved by the McMaster Animal Research Ethics Board.

### CELL CULTURE PREPARATION

Primary hippocampal neurons were generated as previously described by our laboratory with minor modifications (Jacobs and Doering, 2010). Briefly, four embryonic day 15–17, E15–17 (day of sperm plug counted as E1) pups were randomly removed from the pregnant dam. Hippocampi were dissected in calcium and magnesium-free Hank’s buffered salt solution (CMF-HBSS) and tissues were digested with 2.5% trypsin for 15 min in a 37°C water bath. The supernatant was removed, rinsed with three successive washes of CMF-HBSS and re-suspended in neural growth media (NGM) containing 1X Neurobasal (Life Technologies), 0.5 mM GlutaMAX (Life Technologies), and 2.0% B-27 Supplement (Invitrogen). Cells were subsequently plated on 12 mm glass coverslips (Bellco) in 24 multi-well plates, pre-treated with 1 mg/mL poly-L-lysine (Sigma) and 10 μg/ml laminin (Life Technologies), immediately after dissociation at a density of 16,000 cells per well. Neurons were subsequently incubated at 37°C with 5% CO_2_ and remained in culture for 17 days *in vitro* (*DIV*) to allow for the development and maturation of dendritic spines. Every 3–4 days, the neurons were fed by replacing one half of the media with fresh NGM.

### DiI LABELING PROCEDURE

Dendritic spines were identified using the well-characterized fluorescent marker DiI. Application of the dye was adapted from established protocols (Westmark et al., 2011). The neurons were fixed with freshly prepared 2.0% paraformaldehyde (PFA) for 15 min. Each well was gently washed with Dulbecco’s phosphate-buffered saline (DPBS, Invitrogen). For the staining, the wells were aspirated and sprinkled with solid DiI crystals (Life Technologies-Molecular Probes, Cat. #D-3911). Approximately 2–3 crystals were added using a pair of fine forceps to each well. To prevent dehydration of the cells, a small amount of DPBS was dispensed to the edge of the wells. Special care was taken to deliver the smallest crystals to prevent clumping of the dye. The neurons were exposed to the crystals for 10 min on an orbital shaker set at a low speed. The shaker motion ensured that the crystals were adequately distributed to augment complete staining across the surface of the coverslip. The plate was then removed from the shaker and the wells were copiously washed with DPBS to remove all crystals. This procedure was repeated until no crystals were visible. The cells were incubated with DPBS in the dark overnight at room temperature to allow for the diffusion of the dye. The following day, the coverslips were rinsed three times with dH_2_0 for 5–10 min each. The coverslips were removed, completely air-dried, and mounted on slides with prolong gold antifade (Life Technologies – Molecular Probes). Coverslips cured for a minimum of 24 h to allow the liquid mountant to form a semi-rigid gel. Cells were visualized after 72 h from the time of the initial staining to allow the dye to fully migrate across neuronal membranes and diffuse throughout the neurons to highlight spine structures. All images were taken within 7–10 days after coverslipping to minimize fading.

### CONFOCAL IMAGING

Visual imaging of the dendritic spines was acquired using a Zeiss 510 confocal laser-scanning microscope (LSM 510). All images were taken using a 63×/1.2 water immersion lens. A 543 nm Hene-1 Rhodamine laser was utilized to visualize the fluorescence emitted by DiI. To view the specimen with reflected fluorescent light, the reflector turret was programmed to position F set 15 in correlation to the Rhodamine laser, and the single-track configuration was chosen. We used 1024 × 1024 pixels for image size and set the scan speed at a setting of 4. Scan direction and line averaging were also adjusted to a setting of 4. The pinhole diameter was configured to 1 Airy unit (124 μm). Series stacks were collected from the bottom to the top covering all dendrites and protrusions, with an optical slice thickness of 0.5–1 μm. The resulting images (4–6) were then reconstructed to identify hidden protrusions according to Z-stack projections of the maximum intensity.

### DENDRITIC SPINE ANALYSIS

ImageJ software (http://rsbweb.nih.gov/ij/) was used for viewing the confocal images and for spine quantification. In order to increase the magnification for a better view of the spines without loss of image quality, the resolution of the stack image was increased by a factor of 5 in the X and Y directions with the plug-in Transform J Scale (Pop et al., 2012). The length of a spine was obtained by drawing a line from its emerging point on the dendrite to the tip of its head. Approximately, 8–10 neurons selected at random were analyzed per condition across two coverslips. Density and morphology of spines were scored in dendritic segments 10 μm in length. Spines were classified into one of the four morphological subtypes: filopodial, thin, stubby, and mushroom-shaped.

### STATISTICAL ANALYSIS

Spine density was determined by summing the total number of spines per dendritic segment length and then calculating the average number of spines per 10 μm. These values were then averaged to yield the number of spines per 10 μm for each animal. Statistics were performed using GraphPad Prism. Differences were detected with a one-way analysis of variance. Following one-way ANOVA, *post hoc* differences were resolved using the Tukey’s multiple comparison test. A *p*-value of <0.05 was considered significant. All values are expressed as mean ± SEM.

## RESULTS

### LABORATORY PREPARED VERSUS COMMERCIAL GRADE PARAFORMALDEHYDE FIXATION

To determine an optimized protocol for the fluorescent visualization of dendritic spines with the carbocyanine dye in dissociated cultures, we explored patterns of DiI labeling in neurons fixed with laboratory prepared paraformaldehyde (PFA) in PBS or commercial grade formalin at 2.0%. When the cells were prepared with either fixative, the DiI crystals diffused efficiently along the neuronal membranes to permit the effective visualization of the somas and dendritic processes studded with delicate spinous protrusions. However, the laboratory prepared PFA samples facilitated enhanced staining and clarity for the crisp visualization of spines compared to formalin. Commercial grade formalin which typically contains ~10–15% methanol prevents polymerization in storage. Given that DiI is soluble in organic solvents, the use of methanol or acetone fixation is highly discouraged. Taking this into consideration, we investigated whether neurons fixed with acetone would be ineffectively labeled with DiI. We were able to confirm that cells had adhered to the coverslip (as visualized by DAPI) when fixed with acetone, but as expected, the dye unsuccessfully permeated throughout the dendritic segments (results not shown). Additionally, it is important to note that the use of any fixative stored for extended periods of time may risk decomposition and in turn yield poor fixation of samples.

### VARIATIONS IN PARAFORMALDEHYDE CONCENTRATION ALTER CLARITY

**FIGURE 3:**
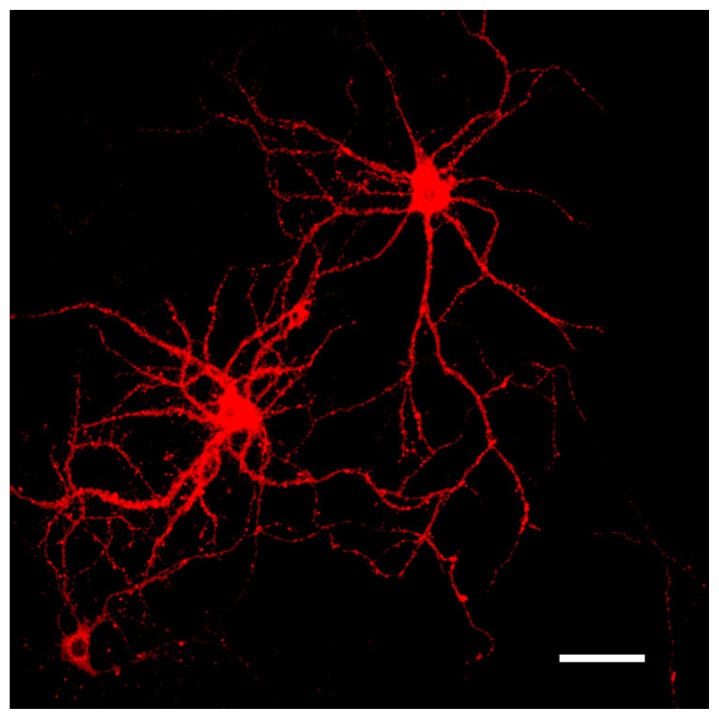
**Representative images of DiI labeled neurons.** DiI highlighting the dendritic complexity and topographical connectivity of neurons. Dendritic arbors and spines are sufficiently filled and visualized in their entirety with the fluorescent dye. Scale bar = 50 μm.

**FIGURE 4:**
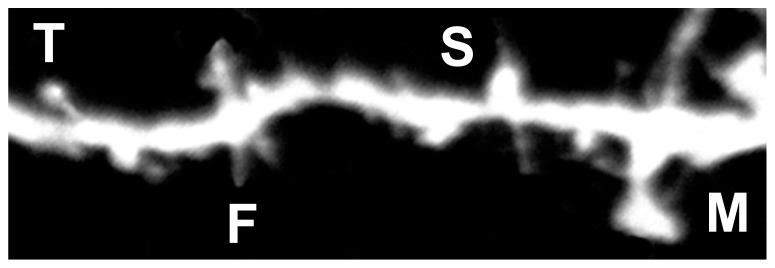
**Representative image of DiI labeled spines.** Filopodia-like (F), long-thin (T), stubby (S), and mushroom (M) spines are identified based on structural measures.

**FIGURE 5:**
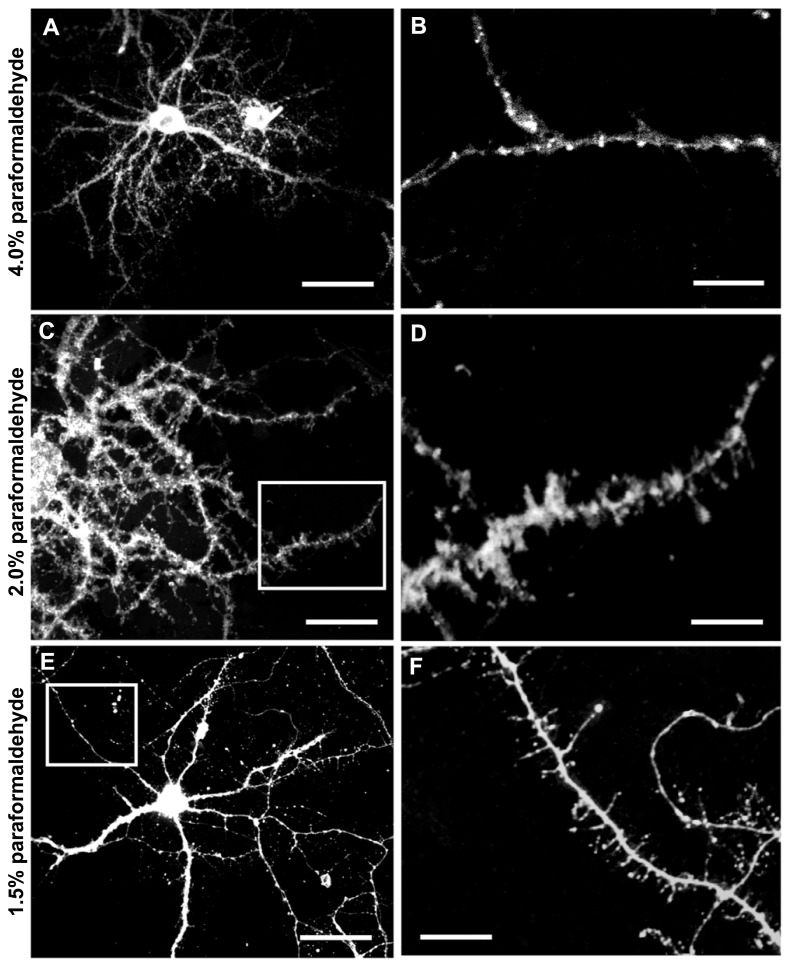
**DiI labeled neurons fixed with varying concentrations of paraformaldehyde (PFA).** Application of DiI crystals on fixed cells produces a very high degree of detail. **(A)** Cell fixed with 4.0% PFA. **(B)** The use of 4.0% PFA fixative significantly compromises DiI diffusion through the dendrites. Multiple varicosities are highlighted along the dendrite and spines are poorly resolved. **(C)** Cell fixed with 2.0 % PFA. **(D)** The use of 2.0% PFA generates high quality DiI labeling of spines. Various subtypes of spines are evident along the dendritic segment. (**E)** Cell fixed with 1.5% PFA. **(F)** The use of 1.5% PFA yields comparable results to the use of 2.0% fixative. Fine, thin filopodial projections are resolved. Scale bar **(A,C,E)** = 50 μm; Scale bar **(B,D,F)** = 5 μm.

Labeling neurons with the fluorescent marker DiI provides a well-defined outline of neuronal cell bodies, dendritic arbors (**Figure 3**), and spine subtypes (**Figure 4**). To determine the most effective conditions for optimal DiI diffusion along dendritic segments, we tested varying concentrations of laboratory PFA fixative at 1.5, 2.0, and 4.0%. Qualitative analysis revealed that the structural integrity of dendrites could not be maintained with a higher concentration of fixation. Namely, the extent of dendritic branching visualized by the dye in cells fixed with 4.0% PFA was hindered compared to cells fixed using 1.5 or 2.0% PFA (**Figure 5A**). In some cases, dye diffusion was limited to where the dye was applied, such that distal dendrites and spines on the same neuron were often not stained, including other neighboring neurons. Swelling of the dendrites (varicosities) were also apparent often hindering accurate measurements of the spines (**Figure 5B**). For instance, the dye would aggregate along the dendrites at spines, causing them to appear “stubby” in shape. However, this often yielded a false morphological classification, as the shape or appearance of spines was attributed to the dye’s inability to completely diffuse throughout the neuronal processes. Furthermore, higher concentrations of PFA typically yielded autofluorescence, which may explain the diffuse background fluorescence coupled with reduced illumination of the spines evident in **Figure 5B**. Ultimately, we discovered that laboratory prepared PFA utilized at lower concentrations was most effective, as it encouraged complete filling of the dendritic segments and finer processes through rapid dye diffusion. Specifically, the images obtained with 1.5–2.0% PFA delineated fine dendritic spines in comparable detail to the traditional Golgi staining method (**Figures 5C–F**). Hence, optimal fixation can greatly improve the quality of DiI neuronal labeling.

### QUANTITATIVE ANALYSIS OF SPINE DENSITY AND MORPHOLOGY

**FIGURE 6:**
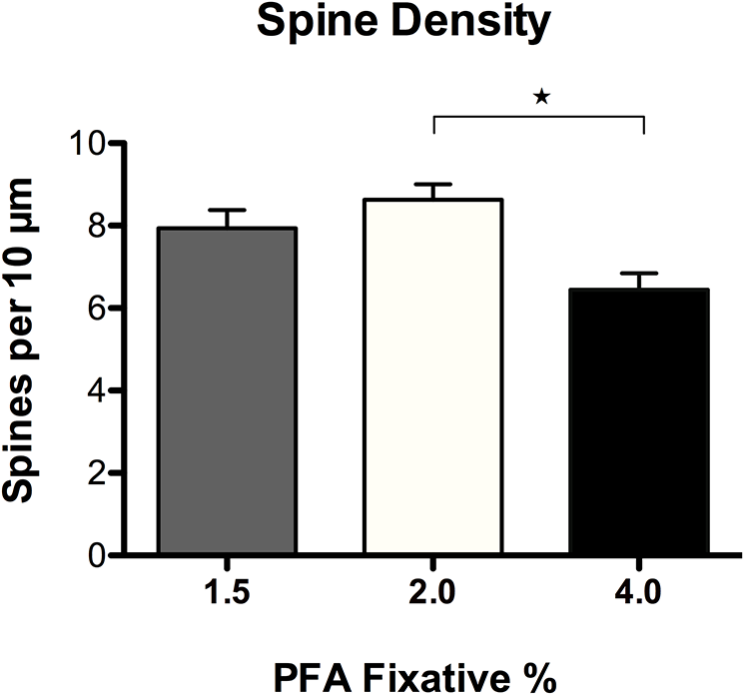
**Spine density analysis of DiI labeled neurons fixed with varying concentrations of paraformaldehyde (PFA).** No significant differences in spine density were observed in neurons fixed with 1.5% or 2.0% PFA. Neurons fixed with 2.0% PFA yielded significantly higher spine densities when compared to neurons fixed with 4.0% PFA (*p < 0.05).

Spine density and morphology were assessed in DiI labeled neurons fixed with varying concentrations of PFA (1.5, 2.0, and 4.0%) to investigate dye permeability. No significant differences in spine density were observed in neurons fixed with either 1.5 or 2.0% PFA (**Figure 6**). Alternatively, neurons fixed with 2.0% PFA yielded significantly higher spine densities when compared to neurons fixed with 4.0% PFA (^*^p = 0.012), potentially as a result of increased DiI labeling. These findings suggest and reinforce our observation that the use of a stronger fixative hinders the dye’s ability to completely diffuse and fill fine processes, like spines. Notably, despite the appearance of an increased proportion of “stubby” spines in neurons fixed with a higher concentration of fixative (**Figure 5B**), no significant differences resulted in any of the comparisons made in the composition of spine morphologies with varying concentrations of fixative (results not shown). Still, our recommendation holds that initial fixation with milder concentrations of PFA fixative at 1.5–2.0% generates the most consistent and superior results.

### SUMMARY OF FINDINGS

The results obtained through this protocol demonstrated that DiI staining in cells prepared with a lower percentage of fixative yielded the highest quality of images. The detailed images generated by this protocol allow us to perform an accurate quantitative analysis of spine structures and spine density. Stubby and mushroom shaped dendritic spines were most evident by their prominent “pinhead” fluorescence directly on the dendritic spine when positioned perpendicularly to the plane of focus on the microscope slide, or as thick protrusions off the dendrite. Filopodial dendritic spines were most visible when their characteristic long and thin protrusions extended upward/downward from the dendritic branch. The high-resolution images that can be obtained using this technique allow us to delineate spine morphologies to provide insight into the areas of synapse formation, development, and remodeling in the CNS.

## DISCUSSION

Several methods to study neuronal structure include histological stains, immunocytochemistry, electroporation of fluorescent dyes, transfection of fluorescent constructs, and the Golgi technique. Although the Golgi technique offers valuable results, this method is time consuming and often lacks reliability. DiI fluorescence labeling has gained popularity, but optimization of the method is essential to accurately quantitate and evaluate fine neuronal structures such as dendritic spines. In lieu of the DiOlistic literature, reported protocols differ vastly for cell/tissue fixation, dye delivery, and diffusion times, with no report on the impact that these different conditions have on the quality of labeling. Here, we outlined a procedure that allows the direct application of DiI to cells in culture; a method that has not been thoroughly explored. The present protocol sought to define the optimal conditions for the fluorescent illumination of individual neurons, including the soma, dendritic arborizations, and spines in cell culture through the use of confocal microscopy. Confocal microscopic analysis of fluorescently labeled neurons has improved resolution of dendritic morphology and has been suggested to provide a more accurate measurement of spines (Lee et al., 2009; Schmitz et al., 2011). Among the most important parameters of this procedure, fixation properties impacted the success of labeling most profoundly.

### OPTIMIZATION OF CELL FIXATION

Amid the DiOlistic literature, a variety of fixation conditions have been reported that produce acceptable levels of DiI labeling. The use of 4.0% PFA is most commonly reported by standard immunohistochemical and immunocytochemical protocols, while many DiI labeling protocols indicate the use of both 1.5 or 4.0% PFA (i.e., Kim et al., 2007; Staffend and Meisel, 2011b; Westmark et al., 2011). To explore this range, we compared the image quality of neurons obtained from 1.5, 2.0, and 4.0% concentrations of fixative. The use of 4.0% PFA fixative significantly compromised DiI diffusion through the dendritic processes (**Figure 5B**). This was apparent by reduced image quality due to increased background fluorescence and inconsistent labeling. However, fixation with both 1.5 and 2.0% PFA yielded similar results with superior diffusion of the lipophilic dye DiI along the neuronal membranes (**Figures 5C–F**). We determined this to be a significant finding since 4.0% PFA has been reported to yield successful results in both tissue slices and cell culture labeling. We discovered that a milder concentration of PFA fixative increased the extent of DiI diffusion, resulting in complete visualization of dendrites and spines. A higher concentration of fixative may interfere and prevent the dye from penetrating the membrane. We strived to further augment our cell fixation technique through a direct comparison of the effectiveness of using freshly prepared laboratory PFA versus commercially produced formalin. Utilization of 1.5–2.0% laboratory PFA produced images with higher clarity and less background fluorescence. The use of commercially -produced formalin (containing methanol) may have adversely impacted the labeling. When evaluating spine density, we found that milder fixation conditions with 2.0% PFA more effectively incorporated DiI into the spines than their 4.0% PFA counterpart. With the use of a weaker fix, more spines were resolved and identified, resulting in an increase in spine density. Together, our results suggest the use of 1.5–2.0% freshly prepared buffered PFA as the superior fixative. As a precaution, utilizing 2.0% (rather than 1.5%) PFA fixative ensures that the cells are effectively fixed and adhered to the coverslip.

### DENDRITIC ORDER OF ANALYSIS

To quantitatively analyze the dendritic spines, we utilized confocal imaging of the cells. The resolution obtained with the confocal microscope permitted the study of individual spines. However, Z stack images with a step interval of 0.5–1 μm supplemented each XY image for accurate spine quantification. The sides of the dendrite were meticulously examined for vertical protrusions stretching upward and downward off the dendrite in addition to the spines extending upward toward the observer. At higher magnification, we were able to morphologically classify some of the dendritic protrusions. However, the spines that extended vertically toward the observer were difficult to morphologically classify from an aerial perspective. Additionally, extra care was taken while examining possible protrusions positioned on the dendritic trunk that extended vertically upward toward the observer; filopodia and thin shaped spines were likely to exhibit less fluorescence in comparison to stubby and mushroom-shaped counterparts, due to less absorption of the dye with reduced surface volume. This point was particularly significant in accurately deciphering the morphology of spines. Moreover, the aggregation of overlapping dendritic processes and complex branching/arborization at times hindered the accurate evaluation of spines. For instance, the areas of the dendritic trunk located closer to the soma-exhibited extreme intertwining with numerous surrounding dendrites that complicated the isolation and differentiation of spines. Additionally, swellings of the dendritic trunk tended to conceal the presence of shorter spines even with a detailed analysis using a series of Z stack images.

Providing that spine density may vary across different order dendrites (primary, secondary, tertiary, etc.), systematic sampling of dendritic order is necessary when analyzing spine density and morphology. Branch ordering schemes are frequently used, wherein the dendrites emerging from the cell soma are primary, their first branches are secondary and so on, with increasing order until the tips are reached. Branches may also vary in diameter. The total number of images collected will depend on the experimental requirements and the degree of variability within a neuron, and across neurons and animals (Ruszczycki et al., 2012). For the purpose of this study, image collection was restricted to the three-dimensional structure of secondary and tertiary dendrites. These parameters were consciously considered to improve accuracy in conducting spine measurements (Rosenzweig and Wojtowicz, 2011). Differences in spine measurements on different order dendrites could potentially draw further insight on neural connectivity in the brain.

### OPTIMIZATION OF DiI DELIVERY

Less than optimal cellular DiI labeling can be attributed to a variety of sources. For troubleshooting purposes, the most common problems, probable causes, and solutions are outlined in **Table 1**.

**Table 1:** Troubleshooting guide for optimal DiI labeling.

Problems	Potential causes/corrective measures
High background is visible.	Residual crystals will result in high background. Avoid adding a surplus of DiI crystals. An excess of crystals will yield high autofluorescence and debris in the cultures. Ensure that coverslips are rinsed well with dH_2_0 until no DiI crystals are visible to the naked eye. Lastly, it is desirable to limit the duration of exposure of the sample to the laser to minimize the degree of phototoxic damage to the ultrastructure and any non-specific signal.
Dye bleeds upon exposure to light.	Glycerol-based mounting media (i.e., Prolong Gold, Vectashield, etc.) can extract membrane-bound dyes upon exposure to light. DiI is light sensitive and long-term exposure will cause fluorescence to fade. Higher magnification objectives (i.e., 63×) are necessary to produce better image resolution and enhance sensitivity of spine detection; however, samples are subject to increased light exposure. High intensity light renders the dye to photobleaching. Minimize duration of light exposure if possible.
Slides are fading.	Ensure that images are captured as soon as possible after mounting. Illumination with light will cause fluorescence to diminish. Slides can be used at least 6 months to a year if stored in the dark at 4°C.
Coverslips appear cloudy.	Ensure that coverslips are rinsed well with dH_2_O or salt residue/film will accumulate clouding the coverslip. Apply more washes if necessary.
Bubbles are apparent after mounting.	Avoid the formation of air bubbles. Ensure that coverslips are completely dry before mounting. Do not apply an excess of mounting medium. Apply a small amount using a dropper to the coverslip and gently pick up the coverslip using the slide. As the coverslip pulls against the slide, allow the mountant to gradually permeate without applying additional pressure.
Absence or lack of cells present.	Fixation of cells may have been unsuccessful. Higher concentration of fixative may be required if cells are not adhering to the coverslip. Always use freshly prepared fixative. Avoid rigorous washes that may cause cells to lift.
Low frequency staining of neurons.	Due to the dye’s indiscriminate nature, this technique often generates sparse fluorescent labeling. During the application, DiI crystals must be thoroughly dispersed to maximize the staining of cells. High concentration of fixative may also obstruct dye diffusion. Do not extend the duration of fix, as it will affect labeling. Overfixation will disrupt the cell membrane integrity causing DiI to leak out of the cell.
Streaking across coverslips.	Scratching of the coverslip with the glass pipette during extraction of solutions from the well impacts image quality. Since this procedure involves numerous washes, it is important to slowly add or remove solutions from the wells to prevent lifting of the cells. One can practice gentle pipetting techniques using the sides of the wells to allow solutions to slowly cover the cells. Extract solutions from the side of the well to avoid contact with coverslip and prevent scratching.
Difficulty isolating single neuronal processes for analysis.	Overlapping of cells and processes may be caused by high density. Reduce plating density.
Dendritic spines are poorly resolved.	Confocal imaging parameters may not be optimal for assessing spine morphology. For high-resolution images obtained at high magnification, slower image acquisition should be used. Adjust settings for detector gain, line averaging, and speed of scanning to improve image quality. The same imaging parameters should be used throughout the study.

### POSSIBLE LIMITATIONS OF THIS METHOD

Although this approach has proven to be effective, it is important to address some of the limitations to the technique. For instance, DiI labeling can be highly variable, because dye crystal size, density, and penetration are all very difficult factors to control. Despite the majority of cells being labeled, the application of extracellular solid DiI crystals often restricted complete staining of all neurons in its entirety within a culture well. Dye diffusion was constrained to neurons that were in close proximity to the crystals, whereas more distal neurons or terminal branches were not prominently stained or filled in. As such, this method is most appropriate for the structural analysis of dendritic spines rather than a comprehensive analysis of dendritic arborization. While these methods produce an accurate analysis on a single-cell resolution, extrapolating the acquired data to a larger neuronal population might prove inaccurate if the staining technique selectively labels only subsets of neurons. Additional selection bias might also occur in these cases if the researcher chooses to measure “convenient” cells, which are visualized more clearly and without overlap with other neurons. Therefore, it is of the utmost importance to ensure that cultures are grown at a sufficient density that limits overlapping processes and permits the isolation of single entities (spines). By using the appropriate concentration of fixative, this can ensure that most neuronal processes are appropriately filled in. Additionally, ensuring that the solid dye is thoroughly distributed during the staining procedure will highlight a larger proportion of cells per sample in a well.

As the morphology of dendritic spines is highly variable, a sufficient sampling size is essential for statistical analysis. With light or epifluorescent microscopy, the dendrite may obscure spines that lie above or below the visual plane, such that only spines extending laterally can be accurately counted. However, this problem cannot be completely remedied by three-dimensional confocal microscopy. To compensate, some studies have applied correction factors for hidden spines (Bannister and Larkman, 1995).

It is also desirable to combine DiI labeling with immunofluorescent staining, with which detailed co-localization can be analyzed using confocal microscopy. The two techniques, however, are often incompatible because Triton X-100, a conventional detergent or permeabilization reagent commonly used to enhance antibody penetration into tissues or cells, causes diffusion of DiI from the labeled structures (Neely et al., 2009). Since Triton X-100 solubilizes lipid molecules almost indiscriminately, it is most likely that Triton X-100 compromises the retention of DiI in the cellular membrane. As a result, the dye potentially leaks out of the membrane, causing the label to disappear after immunocytochemical procedures (Matsubayashi et al., 2008). The ability to perform a dual staining would better allow investigators to phenotypically characterize DiI labeled cells. In future studies, it may be valuable to couple DiI labeling with other fluorescent markers and/or antibodies to immunocytochemically identify other target proteins of interest within a neuron. Investigating appropriate fluorescent immunocytochemical protocols compatible with DiI neuronal tracing would serve as a useful tool in advancing current labeling techniques.

### ADVANTAGES OF THIS METHOD

The methods outlined above using DiI labeling offer a reproducible protocol that have several advantages for the analysis of dendritic spine structures using photostable fluorescence. This protocol offers the opportunity to systematically analyze a large quantity of dendritic spines in high detail, which cannot be achieved through other neuronal identification methods. Furthermore, fluorescent staining and imaging by confocal microscopy yields a series of Z stack images. Many densely compacted segments and spine protrusions often do not lie favorably in the plane of focus and thus cannot be reliably counted. Confocal imaging with DiI labeling permits the sensitive detection of spines by allowing a three-dimensional analysis of spines and dendrites to avoid over and undersaturated pixels. This is particularly vital for the identification of spinous protrusions on the dendritic trunk and most proximal to the soma, and in other cases where there is frequent overlap of the dendrites. Finally, our described methods are simple and do not increase the costs or effort, and more importantly do not compromise the integrity of the neurons or the quality of the staining and data acquired. Taken together, these characteristics make DiI a powerful technique for identifying and studying early events in neuronal development and brain connectivity with significant implications for neurological disease.

## CONCLUSION

Given the literature, a variety of labeling and diffusion conditions can produce acceptable levels of fluorescent DiI labeling. Our goal was to explicitly compare specific methodological components to determine a DiI protocol that produces reproducible staining of dendritic spines in dissociated cultures. Dendritic spines are significant structural substrates for synaptic plasticity and in turn are vital to the proper functioning of the CNS. Spines serve as a functional integrative unit whose morphology is tightly correlated with its function. An accurate neuronal visualization method provides valuable insight into the neuronal organization of various areas of the brain. Importantly, our technique provides an alternative method to fluorescently label neurons and dendritic spines in a convenient and cost-effective manner. Our technique further enables the analysis of dendritic spine topographical distribution, quantitative measurement, and morphological assessment. Such findings would be highly applicable to the investigation of the etiology of various disorders in which spine pathology has been implicated. As a result, this accurate, efficient, and economical staining technique has a wide array of applicability to the study of CNS neurobiology in normal and disease states.

## Conflict of Interest Statement

The authors declare that the research was conducted in the absence of any commercial or financial relationships that could be construed as a potential conflict of interest.
